# Supplementary material for: Immune regulation of neuronal stem cells in the medaka retina
Source: Development. 2026 Sep 14;153(17):dev205198. doi: 10.1242/dev.205198 (PMC13615679; doi:10.1242/dev.205198)
Supplement: Supplementary file 2 [file develop-153-205198_review_history.pdf]

Immune regulation of neuronal stem cells in the medaka retina

Rashi Agarwal, Joergen Benjaminsen, Katharina Lust, Clara Julseth, Natalia Fuchs, Eva Hasel de Carvalho, Fanny Eggeler, Omnia El Said Ibrahim, Narges Aghaallaei, Baubak Bajoghli and Joachim Wittbrodt
DOI: 10.1242/dev.205198

Editor: Steve Wilson

Review timeline

Original submission:	27 August 2025
Editorial decision:	9 October 2025
First revision received:	9 January 2026
Editorial decision:	12 February 2026
Second revision received:	15 July 2026
Accepted:	29 July 2026

Original submission

First decision letter

MS ID#: dev.205198

MS TITLE: Immune surveillance and pruning of neuronal stem cells in the medaka retina

AUTHORS: Rashi Agarwal, Joergen Benjaminsen, Katharina Lust, Clara Julseth, Natalia Fuchs, Eva Hasel de Carvalho, Fanny Eggeler, Omnia El Said Ibrahim, Narges Aghaallaei, Baubak Bajoghli and Joachim Wittbrodt

Dear Jochen,

I have now received all the referees' reports on the above manuscript, and have reached a decision. The referees' comments are appended below, or you can access them online: please go to:

As you will see, the referees express considerable interest in your work, but have some criticisms and suggestions for improvements. If you are able to revise the manuscript along the lines suggested, I will be happy to receive a revised version of the manuscript. Please also note that Development will normally permit only one round of major revision. If it would be helpful, you are welcome to contact us to discuss your revision in greater detail. Please send us a point-by-point response indicating your plans for addressing the referees' comments, and we will look over this and provide further guidance.

Please attend to all of the reviewers' comments and ensure that you upload both a 'clean' version of your Word file, along with a highlighted version clearly showing where you have made changes in the revised manuscript. Please avoid using 'Tracked changes' in Word files as these are lost in PDF conversion. I should be grateful if you would also provide a point-by-point response detailing how you have dealt with the points raised by the reviewers in the 'Response to Reviewers' box. If you do not agree with any of their criticisms or suggestions please explain clearly why this is so.

Reviewer 1

SUMMARY OF THE ADVANCE MADE IN THIS PAPER AND ITS POTENTIAL SIGNIFICANCE TO THE FIELD

© 2026. Published by The Company of Biologists under the terms of the Creative Commons Attribution License (https://creativecommons.org/licenses/by/4.0/).

This study addresses an original and, to date, unexplored question regarding the regulation of the retinal stem cell pool by immune cells. Specifically, Agarwal et al. demonstrate that microglia actively phagocytose retinal stem cells in the ciliary marginal zone (CMZ) of the medaka retina, through a mechanism independent of apoptosis. They further show that depletion of microglia results in stem cell overaccumulation, underscoring their role in tissue homeostasis. The authors also identify a chemokine-receptor axis, in which retinal stem cells express ccl25b and microglia express its receptor ccr9a, and establish that this Ccl25b-Ccr9a signaling pathway mediates homeostatic phagocytosis of stem cells. Moreover, following laser-induced injury of retinal stem cells, loss of ccl25b impairs microglial migration toward the injury site in the CMZ.

Overall, this study employs elegant genetic tools combined with advanced 3D imaging, is very well written, and presents largely convincing data accompanied by high-quality figures. This makes an interesting and novel contribution to our understanding of immune surveillance and stem cell maintenance in the retina. That said, a number of issues would benefit from clarification or further discussion, as outlined below.

SUGGESTIONS TO AUTHORS

Major comments

1. The term "pruning" is used at several places, including in the title, but may be misleading. Pruning generally refers to the removal of parts of a cell. Here, however, the authors observed microglia containing H2B-RFP-positive nuclei within their phagosomes, indicating the uptake of stem cell nuclei. This suggests full engulfment and clearance of the stem cell, rather than pruning. The authors should clarify this distinction and/or better define what they mean by pruning.
2. The authors state in their abstract that loss of microglia results in "morphological defects in the retinal stem cell niche." The meaning of "morphological defects" is unclear. Are they referring to growth defects? If so, this should be specified more explicitly, as the current wording is ambiguous.
3. If the stem cell number is partly regulated by phagocytosis, how do the authors explain the absence of a detectable effect on stem cell abundance in ccl25-/- mutants, despite reduced phagocytosis?
4. The legend of Figure 2A refers to "single cells of the dorsal CMZ" showing GFP expression. This phrasing seems to imply a dorso-ventral asymmetry. If there is no such D-V difference, it would be clearer to omit the qualifier "dorsal." In addition, this GFP staining in the CMZ is not visible. There is a GFP-positive cell on the opposite side of the RPE. Is this the signal being described? Please clarify.
5. In the Figure 2A legend, the authors claim that there is also GFP signal in Müller glia. However, without co-labeling using a Müller glia-specific marker, it is unclear how the authors distinguish Müller glia from microglia staining. In addition, the legend should explicitly state that MG refers to Müller glia (which is implied from the cell shape in the schematic).
6. The legend of Figure 2B refers to a "phagocytic cup in the process of forming," but this is not easily discernible in the image, especially for non-specialist readers. Including a schematic illustration of the process would greatly aid interpretation.
7. The manuscript alternates between the terms "immune cells," "macrophages," and "microglia," which is confusing. Since ccr9a-positive cells are identified as microglia in the results, the term "microglia" should be used consistently thereafter.
8. The authors show that microglia depletion increases stem cell number while reducing overall retina size. How do the authors interpret these apparently opposite effects? Is the increase in stem cells due to lack of phagocytosis, or could CMZ expansion be a compensatory response to smaller retinal size? Even if experimental testing is difficult, these hypotheses warrant deeper discussion.
9. Several comparisons were analyzed with a t-test. Given the small sample sizes, a non-parametric test (Mann-Whitney) would be more appropriate.

Minor comments

10. The meaning of the dashed line in Figure 1D should be indicated in the legend.

© 2026. Published by The Company of Biologists under the terms of the Creative Commons Attribution License (https://creativecommons.org/licenses/by/4.0/).

11. At many places, the genotypes "+/-" and "-/-" are not written in superscript.
12. A space should precede parentheses containing references throughout the text.
13. The two sentences are redundant: "Average velocity, migration distance and mean square displacement (MSD) as a function of time were calculated for each immune cell. Average velocity over time and total migration distance were calculated for each individual immune cell."

Reviewer 2

SUMMARY OF THE ADVANCE MADE IN THIS PAPER AND ITS POTENTIAL SIGNIFICANCE TO THE FIELD

In this manuscript by Agarwal et al, the authors address the research question: "How do microglia regulate the retinal stem cell niche?". They identify the specific expression of the chemokine ccl25b in retinal stem cells and of its receptor ccr9a by microglia as potential interacting partners for the retinal stem cell niche. Using transgenic reporters and mutant line in Medaka as model system, the authors show evidence supporting microglia phagocytosis of retinal stem cells. Agarwal et al then use CRISPR/Cas9 to test the impact of the ccl25b:ccr9a signalling axis in regulating this process and show that phagocytosis of retinal stem cells by microglia is reduced in ccl25b mutants in a retinal injury model. To explore the wider relevance of this process, the authors then generated CRISPR mutants that globally lack microglia. These animals exhibit enlarged retinal stem cell pools and aberrant, asymmetric retinal growth (one eye being larger than the other in microglia/macrophage deficient spi1b mutants).

When sufficiently substantiated, this manuscript nicely demonstrates that microglia/macrophages are important for regulating the size of the retinal stem cell pool and control of overall retinal growth. Furthermore, it provides a novel role of ccl25b:ccr9a signalling in mediating phagocytosis of retinal stem cells by microglia. However, it appears that this signalling axis is not required for the phenotypes that the microglia-deficient Medaka fish show. This circumstance slightly diminished my enthusiasm as it left it unclear for which biological process ccl25b is significant for. Is it only for microglial responses following injury? Are there additional mechanism playing a role that lead to redundancies in the uninjured developing animal?

It is clear to me that fully resolving these questions is likely beyond what is realistically achievable here. However, with the manipulations presented, the different results felt quite disconnected when I was reading the manuscript and would benefit from additional experiments to make a coherent story.

SUGGESTIONS TO AUTHORS

- My understanding is that Spi1 is a key transcription factor for many myeloid and lymphoid cells. Are the authors sure that microglial deficiency is the only defect in these animals that can account for the phenotypes reported? Alternatively, use of additional microglial mutants such as irf8 deficient animals would be models that would strengthen the interpretation of their data.

- I totally understand that there may be no requirement for ccl25b:ccr9a signalling due to the presence of additional pathways. However, the authors could address the following: "Does ectopic expression of ccl25b on a different cell typed target cells for microglial phagocytosis?" Such an experiment would greatly strengthen the evidence that this signalling mechanism is relevant in the uninjured retina, even if it isn't required.

- Controls for global phagocytosis rates by microglia could clarify whether this signalling pathway plays a specific role in RSC regulation or a more global effect on microglial activity. i.e. How much RFP is in microglia when expressed by other non-RSC cells, in both the controls and CRISPants?

- It seems that other cells beyond microglia express the ccr9a receptor as shown in figure 2 (i.e. Muller Glia (MG)). Could this receptor expressed by MG also regulate the RSCs?

Another main point is that the discussion is very short and rather flat. The following questions arose to me that are worth discussing:

© 2026. Published by The Company of Biologists under the terms of the Creative Commons Attribution License (https://creativecommons.org/licenses/by/4.0/).

- If less retinal stem cells are phagocytosed in ccl25b mutants but total # stem cells remains the same, are there less RSC proliferative events, faster differentiation or increased apoptosis (without microglial phagocytosis)? By what compensatory mechanism does the stem cell number remain the same?

- Along the same lines, why is there a difference in stem cell # when microglia eliminated vs microglia phagocytosis blocked (ccl25b KD)?

- How could the absence of microglia cause asymmetric retinal growth?

Reviewer 3

SUMMARY OF THE ADVANCE MADE IN THIS PAPER AND ITS POTENTIAL SIGNIFICANCE TO THE FIELD

This study provides evidence that retinal stem cells (RSCs) express ccl25b, while microglia express its receptor ccr9a, and that microglia actively phagocytose RSCs. Disrupting ccl25b reduces uptake of RSC-derived material; conversely, ablating microglia (spi1b knockout) expands the ccl25b$^+$ stem-cell pool. Together, these findings support a model in which microglia prune excess stem cells to maintain homeostasis

SUGGESTIONS TO AUTHORS

This study is timely and promising, however some conclusions are not fully supported by the evidence. More quantification and better markers would strengthen the claims
Mojor points:
1. The authors propose that "ccr9a$^+$ microglia form a concentric double ring in the central CMZ and extend protrusions that directly contact ccl25b$^+$ cells". This organization and the putative contacts are not evident in Fig. 2A-B, and no quantitative analysis is provided. Add higher-resolution/3D evidence with ring completeness and contact frequency, or revise the wording to match the more limited pattern shown.
2. I couldn't locate the video showing microglial engulfment of RSC-derived RFP$^+$ material—has it been uploaded? It may be a platform issue. Since this is central to the study, it would be nice to know more about its dynamics with basic quantification (e.g., time from contact to internalization, % events leading to uptake, n cells/fish/replicates)
3. Do microglia engage more with RSCs than with other retinal cell types?
4. ccl25b/ccr9a-dependent recruitment is shown only after injury. Does it also occur in the absence of injuries? Add data (trajectories in WT vs. ccl25b−/−) or soften the claim.
5. The manuscript would benefit from a clearer validation of microglial loss in spi1−/− retina using microglia-specific markers—e.g., Neutral Red vital staining (Herbomel et al.)—rather than Sudan Black B, which mainly labels neutrophils.

First revision

Author response to reviewers' comments

Point by point response plain font:
Comments from the Reviewers:

Reviewer 1: SUMMARY OF THE ADVANCE MADE IN THIS PAPER AND ITS POTENTIAL SIGNIFICANCE TO THE FIELD

This study addresses an original and, to date, unexplored question regarding the regulation of the retinal stem cell pool by immune cells. Specifically, Agarwal et al. demonstrate that microglia actively phagocytose retinal stem cells in the ciliary marginal zone (CMZ) of the medaka retina, through a mechanism independent of apoptosis. They further show that depletion of microglia results in stem cell overaccumulation, underscoring their role in tissue homeostasis. The authors

© 2026. Published by The Company of Biologists under the terms of the Creative Commons Attribution License (https://creativecommons.org/licenses/by/4.0/).

also identify a chemokine-receptor axis, in which retinal stem cells express ccl25b and microglia express its receptor ccr9a, and establish that this Ccl25b-Ccr9a signaling pathway mediates homeostatic phagocytosis of stem cells. Moreover, following laser-induced injury of retinal stem cells, loss of ccl25b impairs microglial migration toward the injury site in the CMZ.

Overall, this study employs elegant genetic tools combined with advanced 3D imaging, is very well written, and presents largely convincing data accompanied by high-quality figures. This makes an interesting and novel contribution to our understanding of immune surveillance and stem cell maintenance in the retina. That said, a number of issues would benefit from clarification or further discussion, as outlined below.

SUGGESTIONS TO AUTHORS

Major comments

1. The term "pruning" is used at several places, including in the title, but may be misleading. Pruning generally refers to the removal of parts of a cell. Here, however, the authors observed microglia containing H2B-RFP-positive nuclei within their phagosomes, indicating the uptake of stem cell nuclei. This suggests full engulfment and clearance of the stem cell, rather than pruning. The authors should clarify this distinction and/or better define what they mean by pruning.
We agree that pruning usually means part of the cell is removed or phagocytosed to improve cellular function. In this paper, as we observe H2B-RFP nuclei in microglia, it is uncertain whether they are partially phagocytosed. Hence, this term will be avoided in the revised manuscript to provide a clear understanding of the findings.

2. The authors state in their abstract that loss of microglia results in "morphological defects in the retinal stem cell niche." The meaning of "morphological defects" is unclear. Are they referring to growth defects? If so, this should be specified more explicitly, as the current wording is ambiguous.
By morphological defects we mean that the CMZ niche gets bigger in the retina affecting the overall size of the tissue as it grows post-embryonically. In the revised version, we provide supplemental images of retina tissue at a later time point in surviving embryos where morphological changes become apparent as the organism grows. Hence, we keep the term morphological change as it is and provide the supporting context.

3. If the stem cell number is partly regulated by phagocytosis, how do the authors explain the absence of a detectable effect on stem cell abundance in ccl25-/- mutants, despite reduced phagocytosis?
We stipulate that the process of phagocytosis is not completely blocked in the absence of the ccl25b chemokine which is why we don't see any difference in the number of retinal stem cells. In the revised manuscript we take care to phrase this more prominently to avoid a misunderstanding. Like the referee, we had initially been assuming that the ccl25b-ccr9a axis has a direct impact on stem cell numbers, which is not the case. We have taken care to clearly state this important point in the revised version of the manuscript.

4. The legend of Figure 2A refers to "single cells of the dorsal CMZ" showing GFP expression. This phrasing seems to imply a dorso-ventral asymmetry. If there is no such D-V difference, it would be clearer to omit the qualifier "dorsal." In addition, this GFP staining in the CMZ is not visible. There is a GFP-positive cell on the opposite side of the RPE. Is this the signal being described? Please clarify.
We agree with the reviewer that the term dorsal is misleading here. Dorsal was meant in respect to the described CMZ area and not the entire retina. Also, the GFP positive cells in the image are present at the edge of the CMZ so this will be rephrased as follows:
"At the edge of the proximal end of the CMZ"

5. In the Figure 2A legend, the authors claim that there is also GFP signal in Müller glia. However, without co-labeling using a Müller glia-specific marker, it is unclear how the authors

© 2026. Published by The Company of Biologists under the terms of the Creative Commons Attribution License (https://creativecommons.org/licenses/by/4.0/).

distinguish Müller glia from microglia staining. In addition, the legend should explicitly state that MG refers to Müller glia (which is implied from the cell shape in the schematic).
We have assumed that the signals in the INL are detected in MG cells based on the positioning of the cells. To keep this point clear, we adjusted the description and state that the ccr9a expression is also found in the INL.

We additionally support the claim that the cells in INL expressing ccr9a are indeed microglia as we also detect cxcr3a expressing microglia in the INL region of the retina in the supplementary movie 1 describing distribution of microglia throughout the retina.

6. The legend of Figure 2B refers to a "phagocytic cup in the process of forming," but this is not easily discernible in the image, especially for non-specialist readers. Including a schematic illustration of the process would greatly aid interpretation.
As suggested by the reviewer, an illustration for the phagocytic cup has been included in the revised manuscript for better clarification.

7. The manuscript alternates between the terms "immune cells," "macrophages," and "microglia," which is confusing. Since ccr9a-positive cells are identified as microglia in the results, the term "microglia" should be used consistently thereafter.
We thank the reviewers for pointing this out. The term immune cells has been replaced with microglia in the rest of the manuscript following Figure 1. The term macrophages is only used when we discuss the presence of these cells in other tissues as the term microglia is used only in the context of macrophages present in the tissues of the central nervous system.

8. The authors show that microglia depletion increases stem cell number while reducing overall retina size. How do the authors interpret these apparently opposite effects? Is the increase in stem cells due to lack of phagocytosis, or could CMZ expansion be a compensatory response to smaller retinal size? Even if experimental testing is difficult, these hypotheses warrant deeper discussion.
We speculate that the role of microglia in the retina could be controlling the density of cells in the stem cell niche, ensuring proper number of retinal stem cells, which would otherwise lead to overcrowding and block stem cells from differentiating.

9. Several comparisons were analyzed with a t-test. Given the small sample sizes, a non-parametric test (Mann-Whitney) would be more appropriate.
The statistical analysis is changed to the non-parametric test as suggested.

Minor comments

10. The meaning of the dashed line in Figure 1D should be indicated in the legend.
Dashed line has been described as retina.

11. At many places, the genotypes "+/-" and "-/-" are not written in superscript.
The names have been fixed accordingly.

12. A space should precede parentheses containing references throughout the text.
The spaces have been added wherever it was lacking.

13. The two sentences are redundant: "Average velocity, migration distance and mean square displacement (MSD) as a function of time were calculated for each immune cell. Average velocity over time and total migration distance were calculated for each individual immune cell."
Thank you for pointing this out. The second sentence has been removed from the methods section.

Reviewer 2: SUMMARY OF THE ADVANCE MADE IN THIS PAPER AND ITS POTENTIAL SIGNIFICANCE TO THE FIELD

In this manuscript by Agarwal et al, the authors address the research question: "How do microglia regulate the retinal stem cell niche?". They identify the specific expression of the chemokine

© 2026. Published by The Company of Biologists under the terms of the Creative Commons Attribution License (https://creativecommons.org/licenses/by/4.0/).

ccl25b in retinal stem cells and of its receptor ccr9a by microglia as potential interacting partners for the retinal stem cell niche. Using transgenic reporters and mutant line in Medaka as model system, the authors show evidence supporting microglia phagocytosis of retinal stem cells. Agarwal et al then use CRISPR/Cas9 to test the impact of the ccl25b:ccr9a signalling axis in regulating this process and show that phagocytosis of retinal stem cells by microglia is reduced in ccl25b mutants in a retinal injury model. To explore the wider relevance of this process, the authors then generated CRISPR mutants that globally lack microglia. These animals exhibit enlarged retinal stem cell pools and aberrant, asymmetric retinal growth (one eye being larger than the other in microglia/macrophage deficient spi1b mutants).

When sufficiently substantiated, this manuscript nicely demonstrates that microglia/ macrophages are important for regulating the size of the retinal stem cell pool and control of overall retinal growth. Furthermore, it provides a novel role of ccl25b:ccr9a signalling in mediating phagocytosis of retinal stem cells by microglia. However, it appears that this signalling axis is not required for the phenotypes that the microglia-deficient Medaka fish show. This circumstance slightly diminished my enthusiasm as it left it unclear for which biological process ccl25b is significant for. Is it only for microglial responses following injury? Are there additional mechanism playing a role that lead to redundancies in the uninjured developing animal?

The ccl25b signalling axis does not inhibit the phagocytosis of retinal stem cells but reduces it. This is why we think we did not see any change in retinal stem cell numbers as the phagocytosis process still takes place but only by a subset of microglial cells. Additionally, our observations suggest that this signaling affects the microglial mobility under inflammatory conditions. Hence, it does not seem that it is the key mechanism to initiate this phagocytosis process. But the identification of retinal stem cells specifically by the ccl25b chemokine and presence of microglial population by its cognate receptor ccr9a in the retina led us to the finding that the retinal stem cells are regulated by microglial population under homeostatic conditions.

It is clear to me that fully resolving these questions is likely beyond what is realistically achievable here. However, with the manipulations presented, the different results felt quite disconnected when I was reading the manuscript and would benefit from additional experiments to make a coherent story.

SUGGESTIONS TO AUTHORS

- My understanding is that Spi1 is a key transcription factor for many myeloid and lymphoid cells. Are the authors sure that microglial deficiency is the only defect in these animals that can account for the phenotypes reported? Alternatively, use of additional microglial mutants such as irf8 deficient animals would be models that would strengthen the interpretation of their data.

Spi1 also known as Pu.1 is a myeloid precursor cell marker and is predominantly involved in macrophage differentiation. Previous studies in mice and zebrafish have also used knockout models of this factor to study the effects of macrophage absence or depletion. Studying microglia specific markers such as irf8 would also benefit but since macrophages have a diverse population of cells it did not guarantee the absence of microglia from the retina. Hence, we chose a model that affects a broad range of macrophage population.

- I totally understand that there may be no requirement for ccl25b:ccr9a signalling due to the presence of additional pathways. However, the authors could address the following: "Does ectopic expression of ccl25b on a different cell typed target cells for microglial phagocytosis?" Such an experiment would greatly strengthen the evidence that this signalling mechanism is relevant in the uninjured retina, even if it isn't required.

We have expressed Ccl25b in Müller glia cells in a ccl25b mutant background and observed ccr9a+ cells in close proximity to the MG cells.

- Controls for global phagocytosis rates by microglia could clarify whether this signalling pathway plays a specific role in RSC regulation or a more global effect on microglial activity. i.e. How much RFP is in microglia when expressed by other non-RSC cells, in both the controls and CRISPants?

© 2026. Published by The Company of Biologists under the terms of the Creative Commons Attribution License (https://creativecommons.org/licenses/by/4.0/).

This suggestion demands identifying and characterizing cell type specific drivers that allow generating lines that specifically express RFP by non-RSCs. This is potentially interesting but given the limited time for the revision and the generation time of or models system it is out of scope for the revision of this manuscript.

- It seems that other cells beyond microglia express the ccr9a receptor as shown in figure 2 (i.e. Muller Glia (MG)). Could this receptor expressed by MG also regulate the RSCs?
This is an interesting idea. However, MG cells are firmly anchored in the scaffold of the retina while our evidence shows that the ccr9a cells are dynamic and migrating towards the stem cell niche in the CMZ. Hence, it is highly unlikely that the centrally located MG expressing ccr9a can reach the peripheral RSCs in the CMZ.

Another main point is that the discussion is very short and rather flat. The following questions arose to me that are worth discussing:

- If less retinal stem cells are phagocytosed in ccl25b mutants but total # stem cells remains the same, are there less RSC proliferative events, faster differentiation or increased apoptosis (without microglial phagocytosis)? By what compensatory mechanism does the stem cell number remain the same?

We followed the reviewers advice and have extended and deepened the discussion in the revised version of the manuscript. We found no difference in the frequency of apoptosis (number of apoptotic cells per volume) in the ccl25b mutants as shown in Supplementary Figure 3C'. Interestingly, the ccl25b mutants are fully viable and grow normally into adulthood. There are no apparent limitations to viability or fertility under standard laboratory conditions.

- Along the same lines, why is there a difference in stem cell # when microglia eliminated vs microglia phagocytosis blocked (ccl25b KD)?

The ccl25b signaling axis does not block the phagocytosis process. We took care to clearly state this in the revised version of the manuscript. What we observed in the ccl25b mutants (stable mutant line, not a KD) was, as clearly stated, a reduced number of microglia with phagocytosed RSCs (RFP+ material). The number of stem cells was not affected in the absence of the ccl25b chemokine. It is possible that only a subset of microglia is affected by the absence of the chemokine, and the stem cells are phagocytosed more often by another subset of microglia, ensuring proper stem cell numbers. This could explain why we do not observe a change in retinal stem cell numbers. We took care to discuss these points in the revised version of the manuscript.

- How could the absence of microglia cause asymmetric retinal growth?

The absence of microglia is resulting in more retinal stem cells which are distributed anisotropically around the circumference in the CMZ. The resulting anisotropic expansion of the CMZ is not identical in both the retinas which is why we find asymmetrical sizes in the post-embryonic growth where the differences become more apparent with time.

Reviewer 3: SUMMARY OF THE ADVANCE MADE IN THIS PAPER AND ITS POTENTIAL SIGNIFICANCE TO THE FIELD

This study provides evidence that retinal stem cells (RSCs) express ccl25b, while microglia express its receptor ccr9a, and that microglia actively phagocytose RSCs. Disrupting ccl25b reduces uptake of RSC-derived material; conversely, ablating microglia (spi1b knockout) expands the ccl25b^{+} stem-cell pool. Together, these findings support a model in which microglia prune excess stem cells to maintain homeostasis

SUGGESTIONS TO AUTHORS
This study is timely and promising, however some conclusions are not fully supported by the evidence. More quantification and better markers would strengthen the claims
Major points:

© 2026. Published by The Company of Biologists under the terms of the Creative Commons Attribution License (https://creativecommons.org/licenses/by/4.0/).

1. The authors propose that "ccr9a⁺ microglia form a concentric double ring in the central CMZ and extend protrusions that directly contact ccl25b⁺ cells". This organization and the putative contacts are not evident in Fig. 2A-B, and no quantitative analysis is provided. Add higher-resolution/3D evidence with ring completeness and contact frequency, or revise the wording to match the more limited pattern shown.
The concentric double ring arrangement of microglia is provided as a movie in the supplementary material titled: Distribution of microglia throughout retina.
The protrusions are difficult to visualize, but we have seen that microglia move towards the ccl25b+ stem cells, which shows that they do not just extend protrusions but migrate into the stem cell niche (Provided as supplementary movie 3 titled: Microglia interacts with retinal stem cells expressing ccl25b::h2BRFP.) The wording has been corrected in the revised manuscript in this aspect.

2. I couldn't locate the video showing microglial engulfment of RSC-derived RFP⁺ material—has it been uploaded? It may be a platform issue. Since this is central to the study, it would be nice to know more about its dynamics with basic quantification (e.g., time from contact to internalization, % events leading to uptake, n cells/fish/replicates)

We have provided a video where microglia transfer already engulfed RFP+ material to another microglia. As the event and time resolution of capturing the entire process from migration of microglia to engulfment is difficult to capture, we have not been able to quantify the dynamics of this process.

3. Do microglia engage more with RSCs than with other retinal cell types?
This is a valid question. Detailed analysis of the available videos indicates that microglia are mainly migrating towards the stem cells in the CMZ region. This does not exclude that they are also interacting with other cell types.
To unambiguously address the engagement of microglia with other progenitor cell types would require the establishment and validation of the corresponding driver lines and the establishment and validation of transgenic reporter lines. Given the generation time of our model system this cannot be achieved within the remaining time for the revision.
We have taken care to carefully phrase the statements in the revised manuscript and do not claim that the interaction with stem cells is exclusive.

4. ccl25b/ccr9a-dependent recruitment is shown only after injury. Does it also occur in the absence of injuries? Add data (trajectories in WT vs. ccl25b−/−) or soften the claim.
We have softened the claim that the migration ability of microglia was affected under inflammatory conditions by specifying conditions clearly in the results section.

5. The manuscript would benefit from a clearer validation of microglial loss in spi1−/− retina using microglia-specific markers—e.g., Neutral Red vital staining (Herbomel et al.)—rather than Sudan Black B, which mainly labels neutrophils.
We have carried out the Neutral Red vital staining in spi1b-/- and have provided it in the revised manuscript.

Second decision letter

MS ID#: dev.205198R1

MS TITLE: Immune surveillance of neuronal stem cells in the medaka retina

AUTHORS: Rashi Agarwal, Joergen Benjaminsen, Katharina Lust, Clara Julseth, Natalia Fuchs, Eva Hasel de Carvalho, Fanny Eggeler, Omnia El Said Ibrahim, Narges Aghaallaei, Baubak Bajoghli and Joachim Wittbrodt

Dear Jochen,

© 2026. Published by The Company of Biologists under the terms of the Creative Commons Attribution License (https://creativecommons.org/licenses/by/4.0/).

I have now received all the referees' reports on the above manuscript, and have reached a decision. The referees' comments are appended below.

As you will see, referee 2 considers that there are still two important issues that you need to address before publication. Please consider and attend to these two points and the minor suggestions from the third reviewer and ensure that you upload both a 'clean' version of your Word file, along with a highlighted version clearly showing where you have made changes in the revised manuscript. Please avoid using 'Tracked changes' in Word files as these are lost in PDF conversion. I should be grateful if you would also provide a point-by-point response detailing how you have dealt with the points raised by the reviewers in the 'Response to Reviewers' box. If you do not agree with any of their criticisms or suggestions please explain clearly why this is so.

Reviewer 1

The authors have satisfactorily addressed all my previous comments. I have no further comments and I believe the manuscript is suitable for publication.

Reviewer 2

I had raised several major points, most of which could have been addressed experimentally with relative ease. However, it does not appear that any have been added to the new manuscript (at least I do not know where to find them).

- Main point #1 was that I asked how the authors can be sure that the expansion of retinal stem cell numbers in the spi1b mutants is due to the lack of microglia/macrophages and not other immune cells. This is a major result reported by the authors (Figure 4). However, as I wrote, Spi1 is a master transcription factor for the myeloid and lymphoid lineages, meaning that Spi1 mutants including e.g. granulocytes such as neutrophils etc. To substantiate the author's claims, I had suggested to test other mutants where macrophages/microglia are more selectively depleted (e.g. the irf8 mutant PMID: 25615614). Such an experiment could be carried out relatively easily using CRISPants. Alternatively, if the authors think that other major myeloid cells lacking in the spi1b play no roles in the phenotypes that they present here, they could just present data showing their absence from the retina. Unfortunately, I do not see how the revised manuscript addresses my point to strengthen the rigor of their result.

- My point #2 was around the fact that disruption of ccl25b:ccr9a signalling does not reflect the phenotypes observed in the Spi1 mutants in the uninjured retina. I acknowledged that the presence of additional signalling pathways might mask effects mediated by ccl25b:ccr9a signalling (unless the defects seen in the Spi1 mutants are not due to the absence of microglia, see point #1). Hence, I asked for evidence of some type of relevance for this signalling mechanism in the uninjured retina by e.g. ectopically expressing ccl25b on other cell types and to then assess if microglia will target these cells.
The authors reply the following: "We have expressed Ccl25b in Müller glia cells in a ccl25b mutant background and observed ccr9a+ cells in close proximity to the MG cells."
However, I could not find these experiments in the revised manuscript.

Reviewer 3

The authors have addressed most comments by softening their claims and adding clarifications. Although they did not provide requested additional quantitative analyses, their response is satisfactory, as the revised manuscript is now more closely aligned with what the data actually demonstrate. Regarding the title, I agree that the term "pruning" should be removed. I am also not convinced that "surveillance" is appropriate, as the authors ultimately show engulfment of stem cells by microglia. Thus, a title that reflects microglial-mediated engulfment or regulation of stem cells would therefore be more accurate.

© 2026. Published by The Company of Biologists under the terms of the Creative Commons Attribution License (https://creativecommons.org/licenses/by/4.0/).

Second revision

Author response to reviewers' comments

Point by point response to the Reviewers:

Reviewer 1: The authors have satisfactorily addressed all my previous comments. I have no further comments and I believe the manuscript is suitable for publication.

We appreciate the positive feedback and that the revisions have satisfactorily addressed the concerns.

Reviewer 2: I had raised several major points, most of which could have been addressed experimentally with relative ease. However, it does not appear that any have been added to the new manuscript (at least I do not know where to find them).

- Main point #1 was that I asked how the authors can be sure that the expansion of retinal stem cell numbers in the spi1b mutants is due to the lack of microglia/macrophages and not other immune cells. This is a major result reported by the authors (Figure 4). However, as I wrote, Spi1 is a master transcription factor for the myeloid and lymphoid lineages, meaning that Spi1 mutants including e.g. granulocytes such as neutrophils etc. To substantiate the author's claims, I had suggested to test other mutants where macrophages/microglia are more selectively depleted (e.g. the irf8 mutant PMID: 25615614). Such an experiment could be carried out relatively easily using CRISPants. Alternatively, if the authors think that other major myeloid cells lacking in the spi1b play no roles in the phenotypes that they present here, they could just present data showing their absence from the retina.
Unfortunately, I do not see how the revised manuscript addresses my point to strengthen the rigor of their result.

-Point#1: We thank the reviewer for this important suggestion. To directly address the concern that the phenotype observed in spi1b mutants might result from the loss of immune cell populations other than microglia, we performed the requested experiment targeting irf8, a transcription factor that is specifically required for macrophage and microglia differentiation. We generated irf8 crispants by CRISPR/Cas9-mediated targeted mutagenesis and quantified the retinal stem cell (RSC) population. Similar to the phenotype observed in spi1b mutants, depletion of microglia in irf8 CRISPants resulted in a significant expansion of the RSC population. These independent results now demonstrate that the increase in RSC number is not specific to the broader immune defects associated with spi1b loss, but is reproduced by selective disruption of the microglial lineage. This substantially strengthens our conclusion that microglia regulate RSC numbers in the medaka retina. The new data have been incorporated into the Results and Discussion sections and are presented in the new Figure 4.

- My point #2 was around the fact that disruption of ccl25b:ccr9a signalling does not reflect the phenotypes observed in the Spi1 mutants in the uninjured retina. I acknowledged that the presence of additional signalling pathways might mask effects mediated by ccl25b:ccr9a signalling (unless the defects seen in the Spi1 mutants are not due to the absence of microglia, see point #1). Hence, I asked for evidence of some type of relevance for this signalling mechanism in the uninjured retina by e.g. ectopically expressing ccl25b on other cell types and to then assess if microglia will target these cells.
The authors reply the following: "We have expressed Ccl25b in Müller glia cells in a ccl25b mutant background and observed ccr9a+ cells in close proximity to the MG cells."

However, I could not find these experiments in the revised manuscript.

-Point#2: We thank the reviewer for this valuable suggestion. We have now attached the requested experiments testing the consequences of ectopic ccl25b expression in the retina. To this end, we used an rx2 driver, which is active in Müller glia cells as well as retinal stem and progenitor cells of

© 2026. Published by The Company of Biologists under the terms of the Creative Commons Attribution License (https://creativecommons.org/licenses/by/4.0/).

the CMZ and photoreceptors. Transient ectopic expression of ccl25b in an otherwise ccl25b mutant retina resulted in the recruitment of ccr9a-positive microglia to the sites of expression, including Müller glia cells and the CMZ. These findings demonstrate that Ccl25b is sufficient to attract microglia within the uninjured retina and therefore provide direct functional support for the relevance of the ccl25b/ccr9a chemokine axis in mediating microglial localization.

We agree with the reviewer that this is an interesting experiment that further probes the function of the ccl25b/ccr9a signalling axis. At the same time, we would like to note that it extends beyond the primary focus of our study, which is the physiological role of endogenous ccl25b, whose expression in the uninjured retina is restricted to retinal stem cells. The ectopic expression experiments therefore complement, rather than alter, our central findings by demonstrating the chemotactic activity of Ccl25b in vivo.

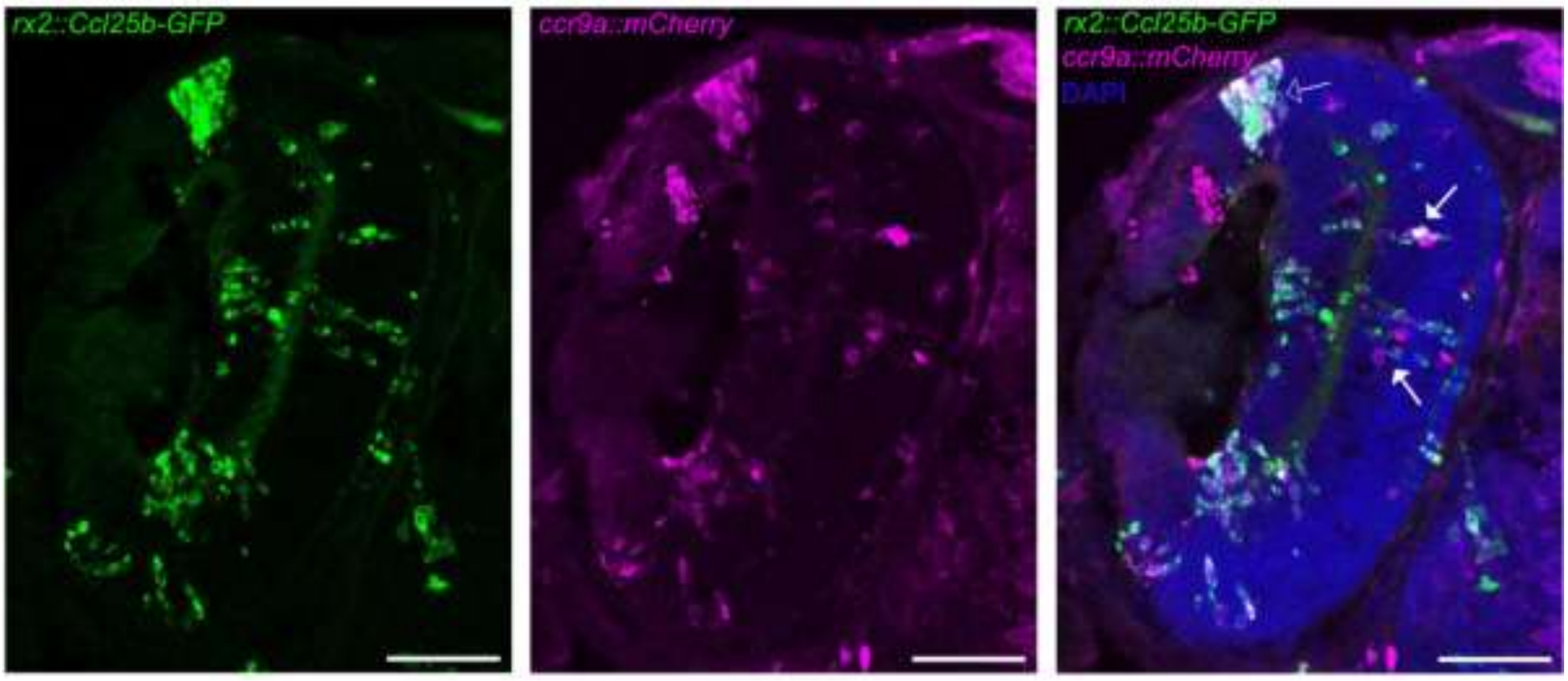

Figure: Co-localization of ccr9a+ microglia and ccl25b-GFP expressing cells in the retina of ccl25b mutants. Ccl25b was transiently ectopically expressed by injection of a rx2::Ccl25b-GFP driver construct into ccl25b-/-;ccr9a::mCherry embryos. Ccr9a+ immune cells (magenta) and ccl25b positive cells co-localize (Müller Glia: white arrowheads) 5 days post-injection. Also note the massive co-localisation in the CMZ (open arrow) further indicating that secreted Ccl25b-GFP attracts microglia (ccr9a::mCherry) to the site of its expression. Scale bar is 50 µm.

Reviewer 3: The authors have addressed most comments by softening their claims and adding clarifications. Although they did not provide requested additional quantitative analyses, their response is satisfactory, as the revised manuscript is now more closely aligned with what the data actually demonstrate. Regarding the title, I agree that the term "pruning" should be removed. I am also not convinced that "surveillance" is appropriate, as the authors ultimately show engulfment of stem cells by microglia. Thus, a title that reflects microglial-mediated engulfment or regulation of stem cells would therefore be more accurate.

*As suggested by the referee, we have replaced the term surveillance in the title with 'regulation'. The new title now reads: "**Immune regulation of neuronal stem cells in the medaka retina**".*

We thank the referees for their constructive comments that helped to improve the statements made in our manuscript.

Third decision letter

© 2026. Published by The Company of Biologists under the terms of the Creative Commons Attribution License (https://creativecommons.org/licenses/by/4.0/).

MS ID#: dev.205198R2

MS TITLE: Immune regulation of neuronal stem cells in the medaka retina

AUTHORS: Rashi Agarwal, Joergen Benjaminsen, Katharina Lust, Clara Julseth, Natalia Fuchs, Eva Hasel de Carvalho, Fanny Eggeler, Omnia El Said Ibrahim, Narges Aghaallaei, Baubak Bajoghli and Joachim Wittbrodt

Dear Jochen,
I am happy to tell you that your manuscript has been accepted for publication in Development, pending our standard publication integrity checks.

Reviewer 2

The authors have now addressed the points that I had raised in my initial review. I have no further comments.

© 2026. Published by The Company of Biologists under the terms of the Creative Commons Attribution License (https://creativecommons.org/licenses/by/4.0/).
